# University of Michigan

**Published:** 1868-07

**Authors:** 


### University of Michigan.

The medical department of the University of Michigan still remains in a very unsettled and unsatisfactory condition; indeed must ever remain an element of contention and discord. It seems to us that State support is not necessary or desirable for a professional school, not desirable or well for either school or State. It is obvious to all experienced observers, that a medical school, situate away from opportunities for clinical study, labors under a fatal disability. Such schools have everywhere fallen to the very lowest point of vitality and are certain to expire. If the State offer almost gratuitous instruction, require no preliminary standard of attainment, and induce young men to graduate in the medical profession because they can do it cheaply and easily, the State must suffer, and the profession must also suffer. While it is well for the State to place general education within reach of the masses, well to found and foster institutions for scientific and literary education, it may now safely leave the professional schools to provide for themselves. Legitimate medicine certainly requires no such aid, and absurd and inconsistent systems of practice would not in the end be gainers by support; it would be no favor to either. We have always believed the medical department of the Michigan University not conducive to the true interests of the profession, and the action of the Legislature has proved this opinion correct. The Faculty were men of the highest merit and their action in promptly resigning their professorships reflects upon them the highest honor; to have retained them would have proved a lasting disgrace. Discord between the Legislature and University can be remedied with advantage to all parties by discontinuance of the medical department, and this seems to be the only desirable and satisfactory manner in which it can be settled.

P. S.—As we go to press we receive and notice the following in the Circular of the Michigan University, and most cheerfully retract what is said above, in any way disparagingly of this school. It is to be hoped that the Michigan Legislature will never again show itself complete fool, in making its appropriations for its educational institutions, and that its past imbecility may not injure the medical department of the University:

"In consequence of an Act of the Legislature of Michigan at its last session, granting aid to the University on the condition that a Professor of homœopathy should be introduced into the Medical Department, much agitation and annoyance have been experienced by its friends; but the Faculty are now happy to announce to the Medical Profession and all the friends of legitimate medicine, that the Board of Regents, who control the University, at a recent meeting resolved, with but a single dissenting vote, that under no circumstances should such professor be introduced into the Medical College at Ann Arbor; and the Supreme Court of the State having since decided that all previous action of the Board making provision for the establishment of a School of homœopathy at another place, is not in compliance with the law, and such action thus becoming null and void, the Faculty are enabled to assure the profession that the *Medical Department of the University of Michigan is entirely free from the remotest connexion with homœopathy*—that its curriculum will not be changed, and that it will remain as heretofore unaffected by any form of irregular teaching or practice."

